# TRPM8-mediated cutaneous stimulation modulates motor neuron activity during treadmill stepping in mice

**DOI:** 10.1007/s12576-019-00707-3

**Published:** 2019-09-03

**Authors:** Kotaro Tamura, Satoshi Sugita, Tadayuki Tokunaga, Yoshihiko Minegishi, Noriyasu Ota

**Affiliations:** 1 Biological Science Research, Kao Corporation, 2606 Akabane, Ichikai-machi, Haga-gun, Tochigi 321-3497 Japan; 2 Personal Health Care Products Research, Kao Corporation, Tokyo, Japan

**Keywords:** TRPM8, Motor neuron, Spinal cord, c-fos, Somatosensory

## Abstract

Motor units are generally recruited from the smallest to the largest following the size principle, while cutaneous stimulation has the potential to affect spinal motor control. We aimed to examine the effects of stimulating transient receptor potential channel sub-family M8 (TRPM8) combined with exercise on the modulation of spinal motor neuron (MN) excitability. Mice were topically administrated 1.5% icilin on the hindlimbs, followed by treadmill stepping. Spinal cord sections were immunostained with antibodies against c-fos and choline acetyltransferase. Icilin stimulation did not change the number of c-fos^+^ MNs, but increased the average soma size of the c-fos^+^ MNs during low-speed treadmill stepping. Furthermore, icilin stimulation combined with stepping increased c-fos^+^ cholinergic interneurons near the central canal, which are thought to modulate MN excitability. These findings suggest that TRPM8-mediated cutaneous stimulation with low-load exercise promotes preferential recruitment of large MNs and is potentially useful as a new training method for rehabilitation.

**Electronic supplementary material:**

The online version of this article (10.1007/s12576-019-00707-3) contains supplementary material, which is available to authorized users.

## Introduction

Motor neurons (MNs) are generally recruited from the smallest to the largest with an increasing load following the size principle [1]. MNs recruited at a low force tend to constitute small motor units (MUs), while large MNs are recruited when higher forces are needed, and they constitute the large MUs [2]. However, there are some exceptions [3]; for example, the order of MU recruitment can be changed by electrically stimulating the cutaneous afferents. The afferent stimulation generates inhibitory responses in the small MUs and excitatory responses in the large MUs [4–6]. These findings suggest that cutaneous afferent input modulates MN excitability via somatosensory reflex pathways. Cutaneous stimulation has the potential to induce adaptive plasticity in the muscles [7] and is applied for the treatment of various neurological and musculoskeletal disorders [8–10].

Skin cooling (SC) induced by a cold environment have been reported to enhance muscle activity during repetitive exercise without changes in the muscle temperature [11]. SC at 25 °C during a slow ramp contraction also changes MU recruitment pattern and selectively induces large MU recruitment [12]. Furthermore, in practical rehabilitation trials, recent studies have shown that maintaining the skin temperature at around 25 °C, using a gel-cooling pad on the quadriceps muscle, enhances muscle activity at 15% maximum voluntary contraction (MVC) [13] and improves the rate of force development (RFD) in the early phase of contraction [14]. On the basis of these studies, it is presumed that maintaining the skin temperature at 25 °C is appropriate for modulating MN excitability without a reduction in the muscle temperature.

Transient receptor potential (TRP) channels play critical roles in the transduction of temperature and a sub-family of M8 (TRPM8) is activated at a temperature of ≤ 25–28 °C [15]. It has been assumed that activation of TRPM8, expressing in the peripheral nerves, is responsible for SC-induced cutaneous input. TRPM8 is also activated by chemical agents, such as menthol and icilin [16–18]. We have previously shown that the application of menthol gel on the skin over working muscles enhances muscle activity at a low load (35% MVC) in both adult and elderly individuals [19]. These findings suggest that TRPM8-mediated sensory input may be responsible for SC-mediated MN excitability. In addition, because the application of menthol gel neither requires any extensive equipment for SC nor limits any movement for subjects, this strategy might be useful to train elderly individuals to recruit large MUs at low-load exercise and to prevent age-related loss of type 2 muscle fibers [20, 21].

However, our previous study assessing MU recruitment with surface electromyography (EMG) could only provide an indication of the activity of the outer layer of the muscle. Therefore, it remains unclear whether TRPM8-mediated cutaneous input in the working muscle modulates spinal MN excitability and induces large MU recruitment. The gene c-fos has been used in identifying activated neurons in the brain and spinal cord during performing various tasks [22, 23]. Previous studies have demonstrated that the number of MNs expressing c-fos increases after a single bout of treadmill stepping in a time- and intensity-dependent manner [24–26]. Detection of c-fos is considered as a marker of neural activity in the spinal cord. The purpose of this study was to investigate the effects of TRPM8-mediated cutaneous stimulation combined with exercise on the modulation of spinal MN excitability. In this study, by using c-fos immunostaining, we found that topical application of a TRPM8 agonist with low-load treadmill stepping promoted c-fos expression in the large MNs, which were supposed to be the fast MNs innervating the type 2 fibers and constitute the large MUs.

## Methods

### Animals

Male C57BL/6J mice (9- to 14-week-old) were purchased from CLEA Japan (Tokyo, Japan) and maintained under a controlled condition (temperature, 23 ± 2 °C; humidity, 55 ± 10%; and lighting, 07:00 to 19:00 h). The mice were provided with standard chow (CE-2; CLEA Japan, Tokyo, Japan) and water ad libitum. All animal experiments were conducted at the Experimental Animal Facility of the Kao Corporation’s R&D Department. The study was approved by the Kao’s Animal Care Committee, and all experiments followed the guidelines of the committee.

### Experimental design

#### Experiment 1

After acclimatization, the mice (*n* = 6) were anesthetized with isoflurane (Abbott Japan, Tokyo, Japan), and the hair of the hindlimbs were shaved by animal clippers. Icilin (QJ-5946, Combi-Blocks, San Diego, CA, USA) was dissolved in 80% DMSO (Wako, Osaka, Japan) and 20% PBS solution, and the concentration was adjusted to 1.5% (w/v). Approximately, 100 μL of the icilin solution was applied on the right hindlimb, whereas a control solution (80% DMSO, 20% PBS) was applied on the left hindlimb of each mouse under isoflurane anesthesia. The solution was gently applied to all parts of the hindlimb thrice with a cotton swab. To provide adequate time for c-fos expression, the mice were returned to their cages and were perfused 90 min after the application.

#### Experiment 2

A 10-lane motorized rodent treadmill (MK-680; Muromachi Kikai, Tokyo, Japan) with no incline was used for treadmill stepping. The experiment was conducted with 3 days of sessions. After shaving the hair from the hindlimbs for the initial 2 days, all mice were accustomed to treadmill stepping at a speed of 10 m/min for 30 min. On the last day, the mice (*n* = 55) were randomly divided into five groups: sedentary (Sed, *n* = 9), sedentary + icilin (Sed + icilin, *n* = 10), low-speed stepping (low-speed stp, *n* = 12), high-speed stepping (high-speed stp, *n* = 12), and low-speed stepping + icilin (low-speed stp + icilin, *n* = 12). The mice were topically administrated with icilin solution or control solution on all parts of the hindlimbs as noted in “Experiment 1”, followed by conducting treadmill stepping or home cage activity. The treadmill stepping was conducted according to the following program: low-speed, 12 m/min for 60 min; and high-speed, 18–20 m/min for 60 min to detect activated neurons as previously described [25]. The stepping speed was set to increase by 2 m/min up to each maximal speed. The mice were returned to their cages and perfused 60 min after the treadmill stepping bout. All mice were kept fasting from 3 h before the treadmill stepping.

### Tissue collection

The mice were anesthetized with isoflurane and perfused transcardially with 20–30 mL of PBS supplemented with 1 unit/mL of heparin sodium (Mochida Pharmaceutical, Tokyo, Japan), followed by 20–30 mL of 4% paraformaldehyde phosphate buffer solution (4% PFA; Wako, Osaka, Japan). The vertebral column containing the spinal cord was dissected and post-fixed in 4% PFA overnight at 4 °C. The spinal cords were carefully dissected from the vertebral column and impregnated with 30% sucrose in PBS for over 3 days at 4 °C. With the dorsal root ganglion of L5 as a landmark, the lower lumbar segments (L3–L5) were embedded in OCT compound (Sakura Finetek Japan, Tokyo, Japan) and stored at − 80 °C.

### Immunohistochemistry

The frozen spinal cord blocks were transversely sectioned to 30 µm thickness by a cryostat. For each mouse, approximately 100–120 of the sections were sampled from the L3 to L5 segment and collected in 12 consecutive groups of 10 free-floating sections in PBS with sodium azide (Rockland Immunochemicals, Limerick, PA, USA). Approximately, 24 sections per mouse, 2 out of 10 sections randomly picked up from each group, were used for immunolabeling in a free-floating manner. The sections were washed in 0.1 M PB and incubated in primary antibodies against c-fos (rabbit, 1:5000, #226 003, Synaptic Systems, Goettingen, Germany) and choline acetyltransferase (ChAT, goat, 1:200, AB144P, EDM Millipore, Temecula, CA, USA) with a diluted solution (1% normal donkey serum, 0.3% Triton X-100, 0.25% λ-carrageenan, 0.01% NaN_3_ in PBS) on a shaker at 4 °C for 3 days. The sections were washed in PBS with 0.3% TritonX-100 (PBS-T) thrice and incubated with secondary antibodies [Alexa 488-conjugated donkey anti-rabbit and Alexa 568-conjugated donkey anti-goat (1:1000, Molecular Probes, Thermo Fisher Scientific, Pittsburg, PA, USA)] with the diluted solution on a shaker for 1 h at room temperature. The sections were then washed in PBS thrice and mounted on Micro Slide Grass (Matsunami Grass, Osaka, Japan). Coverslips were placed with VECTASHIELD Hard-Set Mounting Medium (Vector Labs, Burlingame, CA, USA).

### Analysis of the activated neurons

Digital images were acquired with an all-in-one fluorescence microscope (BZ-X700, Keyence, Osaka, Japan). All images were obtained at a magnification of 10 ×. For quantitative analysis, BZ Analyzer (Keyence) was used to count the c-fos-positive neurons by manual tagging and to measure the soma size of ChAT labeling MN. A ChAT-positive cell merged with c-fos in lamina IX was defined as a c-fos^+^ MN. ChAT-positive cells located in the range of 400 µm width and 300 µm length centering on the central canal were defined as cholinergic interneurons (INs) near the central canal [26] in reference to previous studies of mouse lumbar spinal cord [43, 44], and the number of these cells merged with c-fos was counted. Since immunostaining images of some mice were not quantifiable probably due to inadequate perfusion and fixation, some samples were excluded from the analysis. The numbers of mice in the Sed, Sed + icilin, low-speed stp, high-speed stp, and low-speed stp + icilin groups included in the analysis were 9, 9, 11, 12, and 11, respectively. The quantification was blindly performed by assigning a code number to each mouse and opening the code after all quantification was completed.

### Statistical analysis

All data are represented by the mean ± SD. Group differences between the two groups were tested with unpaired Student’s *t* tests. Moreover, group differences among > 3 groups were tested with one-way ANOVA followed by Tukey’s post hoc test. Comparison of the soma sizes (Fig. 3b) was performed by two-way ANOVA followed by Tukey’s post hoc test. Correlation (Fig. 4c) was assessed by Pearson’s correlation coefficient. The threshold of significance was set at *p* < 0.05. All analysis was performed using Prism 6 statistical software (GraphPad Software, San Diego, CA, USA).

## Results

### Peripheral stimulation with a TRPM8 agonist induces c-fos expression in the spinal cord dorsal horn

**Fig. 1:**
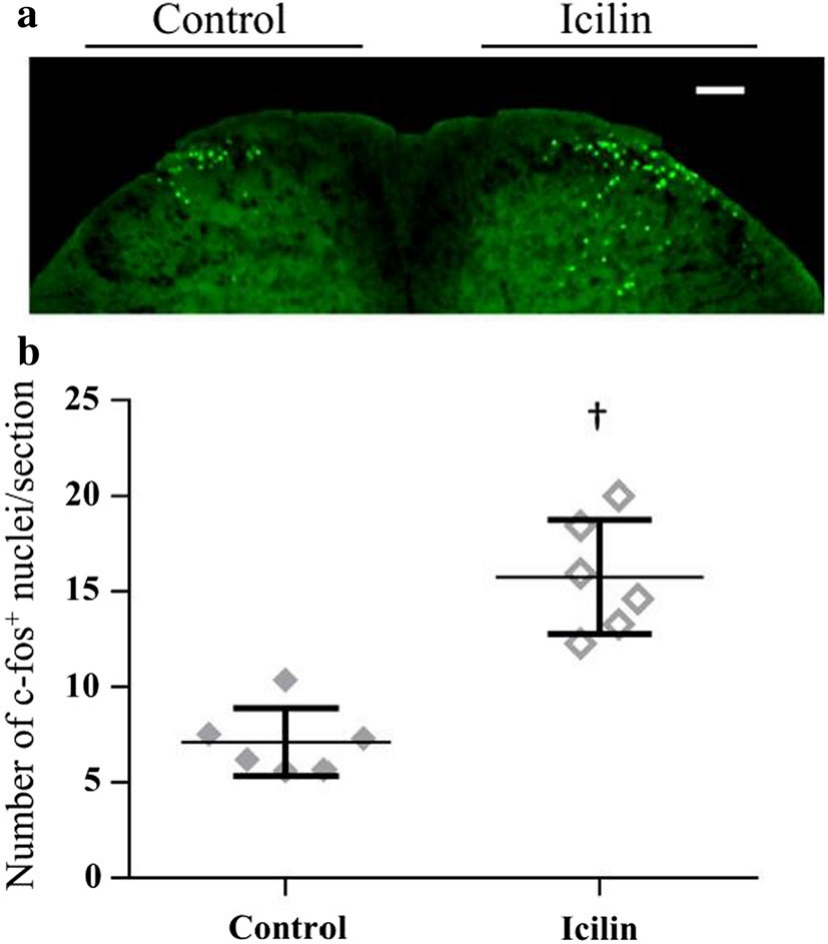
Icilin induced c-fos expression in lamina I and II of the spinal cord dorsal horn. Representative image of the spinal dorsal horn is shown (**a**). Topical application of icilin leads to increased levels of c-fos protein in the L3–L5 region of the ipsilateral dorsal horn (icilin) as compared to that in the contralateral side (control) (**b**). Each symbol represents an individual mouse. The horizontal lines indicate the mean values. Scale bar = 100 μm. Compared with the control, ^†^*p* < 0.01. Data are represented as the mean ± SD

To confirm whether TRPM8-mediated SC stimulation is induced by the cutaneous afferents, we examined the expression of c-fos in the mouse spinal cord dorsal horn following application of a specific TRPM8 agonist, icilin [17] on the hindlimbs. We compared the levels of icilin-evoked c-fos expression to those evoked by the control solvent in lamina I and II of the dorsal horn, as described previously [27]. Application of 1.5% icilin induced significant numbers of c-fos-positive nuclei in the ipsilateral dorsal horn as compared to the contralateral side (Fig. 1). We preliminarily confirmed that 1.5% icilin was enough to induce the c-fos expression in the spinal cord dorsal horn, because the levels were similar to those of 3% icilin, a maximum dissolved concentration (data not shown). Thus, we decided to  use 1.5% icilin in the following experiment.

### TRPM8-mediated cutaneous stimulation modulates c-fos-positive MNs following treadmill stepping

**Fig. 2:**
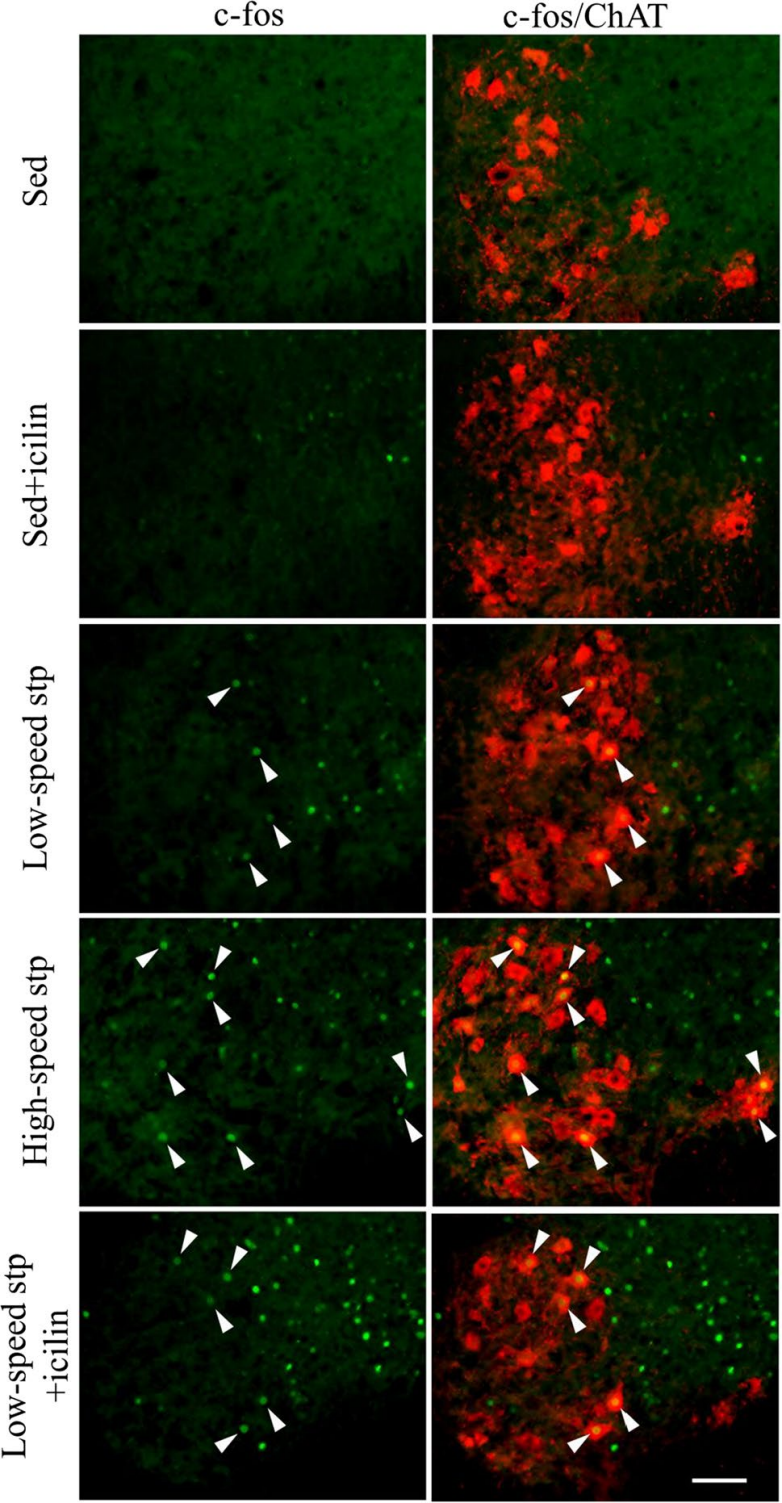
Representative images of the spinal cord ventral horn of each group immunostained with c-fos (green) and ChAT (red) are shown for the sedentary, sedentary + icilin, low-speed stepping, high-speed stepping, and low-speed stepping + icilin groups. C-fos merged in the nuclei of ChAT-positive soma in lamina IX indicates activated (c-fos^+^) MN (white arrowheads). Scale bar = 100 μm (color figure online)

**Fig. 3:**
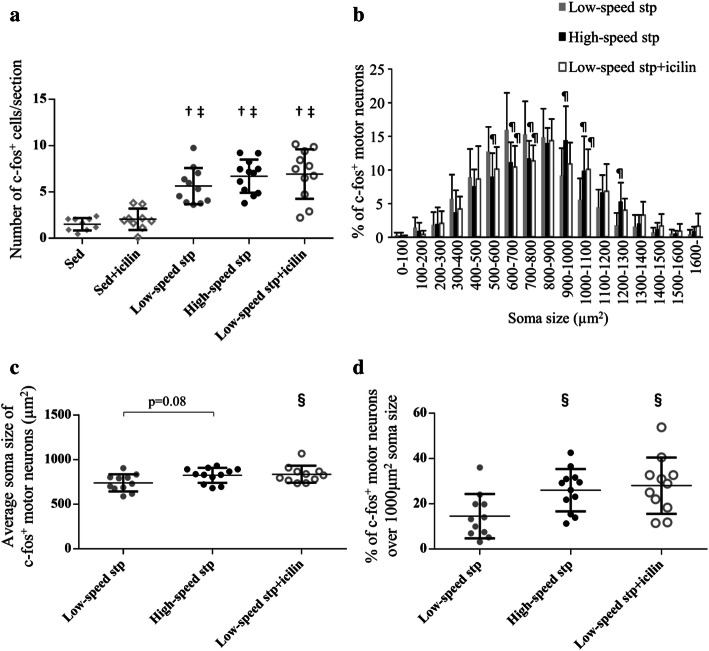
Treadmill stepping with icilin stimulation does not change the number of c-fos^+^ MNs, but modulates the soma size of c-fos^+^ MNs. Quantitative results of c-fos^+^ ChAT-labeled MNs in immunostained images of the spinal cord are shown. The number of c-fos^+^ MN per section is shown for (**a**). Treadmill stepping with icilin stimulation modulates the soma size of c-fos^+^ MNs. The distribution of the c-fos^+^ MNs with respect to their soma sizes (µm^2^) is shown for the stepping groups (**b**). The average soma size of the total c-fos^+^ MNs and the percentage of large soma size (≥ 1000 µm^2^) of the c-fos^+^ MNs are shown in (**c**) and (**d**), respectively. Each symbol represents an individual mouse. The horizontal lines indicate the mean values. Compared with the Sed group, ^†^*p* < 0.01; compared with Sed + icilin group, ^‡^*p* < 0.01; compared with low-speed stp group, ^§^*p* < 0.05, ^¶^*p* < 0.01. Data are represented as the mean ± SD

To evaluate the effects of topical application of icilin with treadmill stepping on the activation of MNs, we defined ChAT-labeled neurons in lamina IX as MNs and observed the c-fos^+^ MNs in the sections of spinal cord ventral horn (Fig. 2). In the quantitative evaluation of c-fos^+^ MNs, the number of c-fos^+^ MNs per section was increased by treadmill stepping, whereas there was no difference in the icilin stimulation or increased stepping speed (Fig. 3a). As the number of c-fos^+^ MNs was unchanged among the low-speed stp, high-speed stp, and low-speed stp + icilin groups, we next evaluated the soma size of c-fos^+^ MNs. The soma size distribution of c-fos^+^ MNs was greatly changed by icilin stimulation or increasing the stepping speed (Fig. 3b). The average soma size of the total c-fos^+^ MNs was also higher in the low-speed stp + icilin group and showed an increasing trend in the high-speed stp group than that in the low-speed stp group (Fig. 3c). We especially focused on the MNs ≥ 1000 µm^2^ of soma size because these neurons are considered to be fast MNs and innervate type 2 fibers [28, 29]. Thus, in the present study, we defined that MNs of ≥ 1000 μm^2^ constitute the large MUs. The percentage of large soma size (≥ 1000 µm^2^) of c-fos^+^ MNs was also higher in the low-speed stp + icilin and high-speed stp groups than that in the low-speed stp group (Fig. 3d). There were few c-fos^+^ MNs observed in the Sed and Sed + icilin groups, and thus we independently showed the size comparison between both groups in Supplemental Fig. 1. The average soma size and the percentage of large soma size of c-fos^+^ MNs were unchanged between the Sed and Sed + icilin groups (Supplemental Fig. 1).

### TRPM8-mediated cutaneous stimulation modulates c-fos-positive cholinergic INs following treadmill stepping

**Fig. 4:**
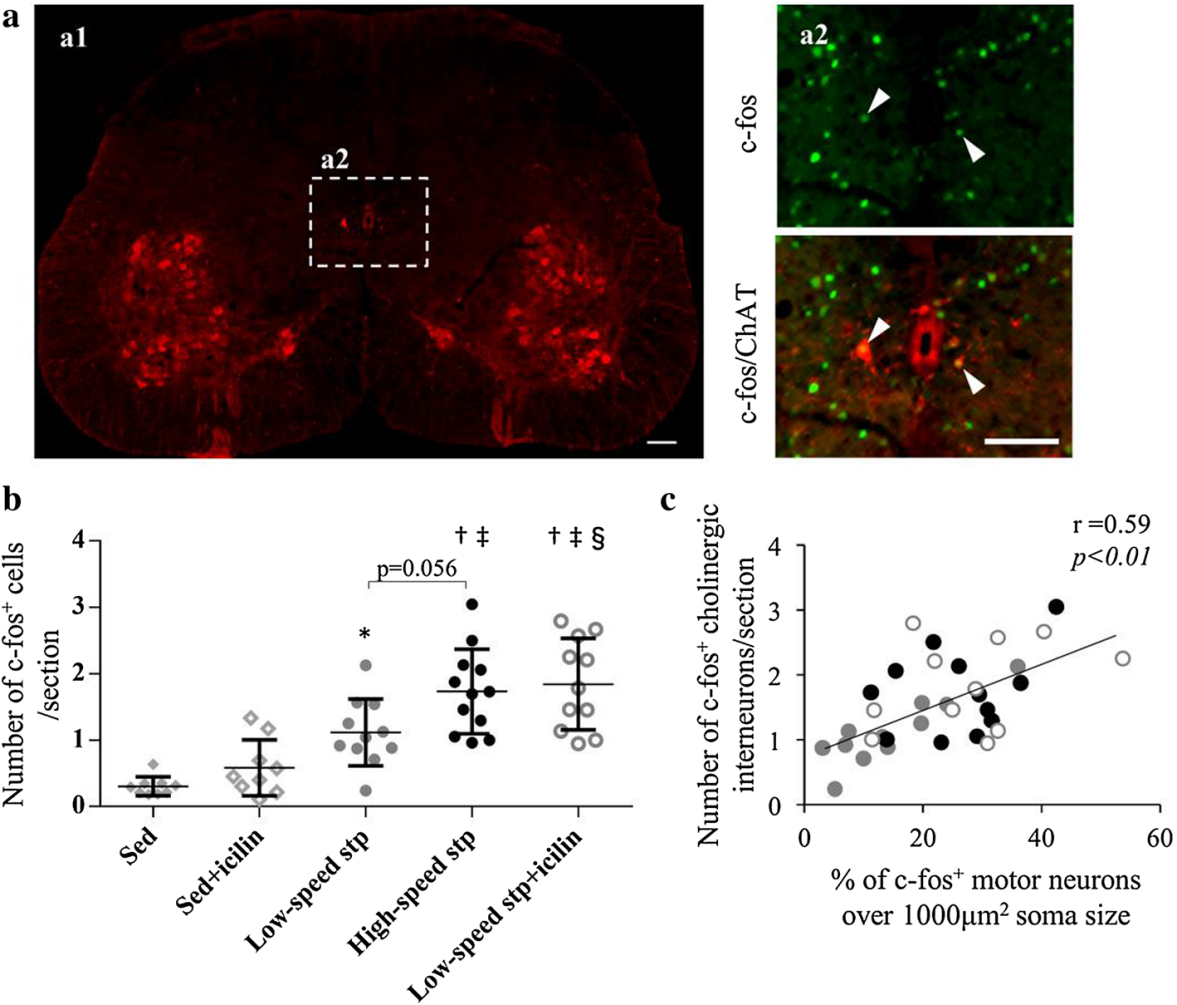
Treadmill stepping with icilin stimulation activates the cholinergic INs. Representative image of ChAT-immunostained spinal cord is shown (a1). Cholinergic INs near the central canal were identified in the range of 400 µm width and 300 µm length centering on the central canal (a2; box with white dashed line). Representative images of the c-fos^+^ cholinergic INs near the central canal (white arrowheads) immunostained with c-fos (green) and ChAT (red) are shown (a2; magnified in the right panels). Scale bar = 100 μm. The number of c-fos^+^ cholinergic INs near the central canal per section is shown for the sedentary, sedentary + icilin, low-speed stepping, high-speed stepping, and low-speed stepping + icilin groups (**b**). Each symbol represents an individual mouse. The horizontal lines indicate the mean values. Compared with the Sed group, *p < 0.05, ^†^*p* < 0.01; compared with the Sed + icilin group, ^‡^*p* < 0.01; compared with low-speed stp group, ^§^*p* < 0.05. Data are represented as the mean ± SD. The correlation between the activation of cholinergic INs and the activation of large MNs in all mice of the three stepping group (*n* = 34) is shown (**c**). Gray circles, black circles, and empty circles with gray outline indicate low-speed stepping, high-speed stepping, and low-speed stepping + icilin, respectively (color figure online)

Spinal interneurons (INs) relay signals between the sensory neurons and MNs. To reveal mechanistic insights on the modulating MN excitability, we focused on the cholinergic INs, which are likely to be involved in sensory processing and motor output [30, 31]. We identified cholinergic INs based on their distance from the central canal (Fig. 4a) as described previously [26, 43, 44]. The number of activated cholinergic INs was higher in the three stepping groups than that in the Sed and Sed + icilin groups (Fig. 4b). This was also higher in the low-speed stp + icilin group and showed an increasing trend in the high-speed stp group than that in the low-speed stp group. Subsequently, we evaluated the correlation between the number of activated cholinergic INs and the percentage of large soma size (≥ 1000 µm^2^) of the activated MNs in each mouse (Fig. 4c). The activation of the cholinergic INs showed a significant positive correlation with the activation of the large MNs.

## Discussion

In this study, we demonstrated that TRPM8-mediated cutaneous stimulation combined with low-load treadmill stepping facilitates the activation of large MNs with the activation of cholinergic INs near the central canal. This provides the mechanistic insight into the previous finding of increased EMG activity during low-load contraction in humans after stimulation of TRPM8 with menthol [19].

We used icilin as a TRPM8 agonist for cutaneous stimulation in mice (Fig. 1). Icilin can cross-activate other TRP channels, such as TRPA1 in vitro [32], while in vivo studies have clearly demonstrated that responses to icilin stimulation in TRPM8^−/−^ mice are reduced to the same extent as that in the control mice [27, 33]. Thus, icilin stimulation is mediated through TRPM8 in vivo. We believed that icilin is percutaneously absorbed and stimulates TRPM8 in the peripheral sensory nerve (Aδ and C-fibers) [34], followed by induction of c-fos expression in the dorsal horn lamina I and II, the primary site of termination of the thermosensitive and nociceptive sensory afferents [35]. Oral or intraperitoneal administration of icilin is reported to produce noxious cold sensations, like wet-dog shakes and jumping behaviors [17, 33], but in our experiments, such behaviors were not observed. The application of icilin to the lower limbs stimulates TRPM8-mediated cutaneous afferents, but not to an extent to induce noxious cold.

We first expected that the number of activated MNs would increase in a stepping speed-dependent manner; however, there was no difference in the results between the low-speed stp and the high-speed stp groups (Fig. 3a). However, the soma size distribution of c-fos^+^ MNs was significantly changed in response to a higher speed (Fig. 3b). It suggested that faster stepping speed leads to suppression of the small MNs and activation of the large MNs, promoting preferential recruitment of the larger MUs. In a limited set of simple contraction, the size principle of rank-ordered MU recruitment clearly holds, while in biomechanically complex movements, MU recruitment has the potential to deviate from the size principle [36]. EMG signals from the hindlimb muscles in rats during treadmill stepping indicate that faster stepping speed leads to preferential recruitment of the larger MUs [37]. It is also reported that the small MUs that are active at low speeds are suppressed, and the large MUs are preferentially activated according to increased exercise speed during bicycling or ankle flexor movements in humans [38, 39]. These findings suggest that the changes in the MU recruitment patterns are flexible to meet the mechanical demands of each task. Therefore, the present results obtained from c-fos immunohistochemistry are consistent with the previous findings obtained from EMG analysis [37–39]. Furthermore, icilin stimulation induced similar c-fos expression patterns with an increased stepping speed (Fig. 3). In spite of no change in the stepping speed, TRPM8-mediated cutaneous stimulation may affect the modulation of spinal MN excitability and induce preferential recruitment of the large MUs. Since the application of icilin on sedentary mice did not change the number and the soma size of c-fos^+^ MNs (Fig. 3a, Supplemental Fig. 1), the modulation of spinal MN is probably induced by a synergistic effect of TRPM8-mediated cutaneous stimulation and treadmill stepping. The modulation of spinal MN by icilin stimulation is possible to affect the running behavior, because SC on the quadriceps muscle improves RFD during isometric knee extension in human research [14]. Quantitative gait analysis of mice treated with icilin is required for future study.

We also found that the number of activated cholinergic INs near the central canal increase following low-speed stepping with icilin stimulation (Fig. 4b). These neurons directly synapse onto MNs and regulate their excitability [31, 40] via cholinergic presynaptic terminals, C-boutons [41]. Acetylcholine (ACh) released from the C-boutons activates M2 muscarinic ACh receptors (M2-mAChRs) on MNs [41] and enhances the MN firing frequency [31]. M2-mAChRs are found to be predominantly expressed in the large MNs, like those innervating mainly the fast medial gastrocnemius muscles [42]. Although the details of innervation by INs are still unclear, these findings suggest that large MNs are likely to be modulated via cholinergic INs. Consistent with this hypothesis, our results show that the activation of cholinergic INs positively correlates with the activation of large MNs (Fig. 4c). A recent study has shown that treadmill stepping in rats activates these neurons by imposing additional loading by increasing the inclination of the treadmill [26]. The authors discuss that increased proprioceptive sensory input by increasing the inclination may affect activation of the cholinergic INs. Therefore, TRPM8-mediated sensory input may affect the proprioceptive sensory input and modulate the motor–sensory feedback during exercise. The other possibility is that the TRPM8-mediated sensory input directly activates the cholinergic INs because icilin stimulation tended to slightly increase the number of activated cholinergic INs in the sedentary mice (Fig. 4b). Although further study is required to elucidate this mechanism, TRPM8-mediated modulation of MN excitability might be regulated, at least in part, by the cholinergic INs near the central canal.

## Conclusion

We found that topical application of a TRPM8 agonist with low-load treadmill stepping promoted preferential activation of large MNs which constitute the large MUs. TRPM8-mediated modulation of the spinal MN excitability may be regulated by cholinergic INs near the central canal. These findings could theoretically support the possibility of using cutaneous afferent input as a new training method for neurorehabilitation. Further studies are needed to examine whether repeated TRPM8-mediated cutaneous stimulation with low-load training promotes muscle fiber hypertrophy or improves muscle strength.

### Electronic supplementary material

Below is the link to the electronic supplementary material.

Supplementary material 1 (DOCX 83 kb)
