# Supplementary material for: TRPM8-mediated cutaneous stimulation modulates motor neuron activity during treadmill stepping in mice
Source: J Physiol Sci. 2019 Sep 3;69(6):931–8. doi: 10.1007/s12576-019-00707-3 (PMC10717255; doi:10.1007/s12576-019-00707-3)

**Supplementary material**

**Supplemental Figure Legends**

**Supplemental Figure 1.** Application of icilin on sedentary mice does not change the soma size of c-fos^+^ MNs. Quantitative results of c-fos^+^ MNs in immunostained images of the spinal cord are shown for Sed and Sed+icilin groups. The average soma size of the total c-fos^+^ MNs and the percentage of large soma size (≥ 1000 µm^2^) of the c-fos^+^ MNs are shown in (a) and (b), respectively. Each symbol represents an individual mouse. The horizontal lines indicate the mean values. Data are represented as the means ± S.D.

**Supplemental Figure 1**


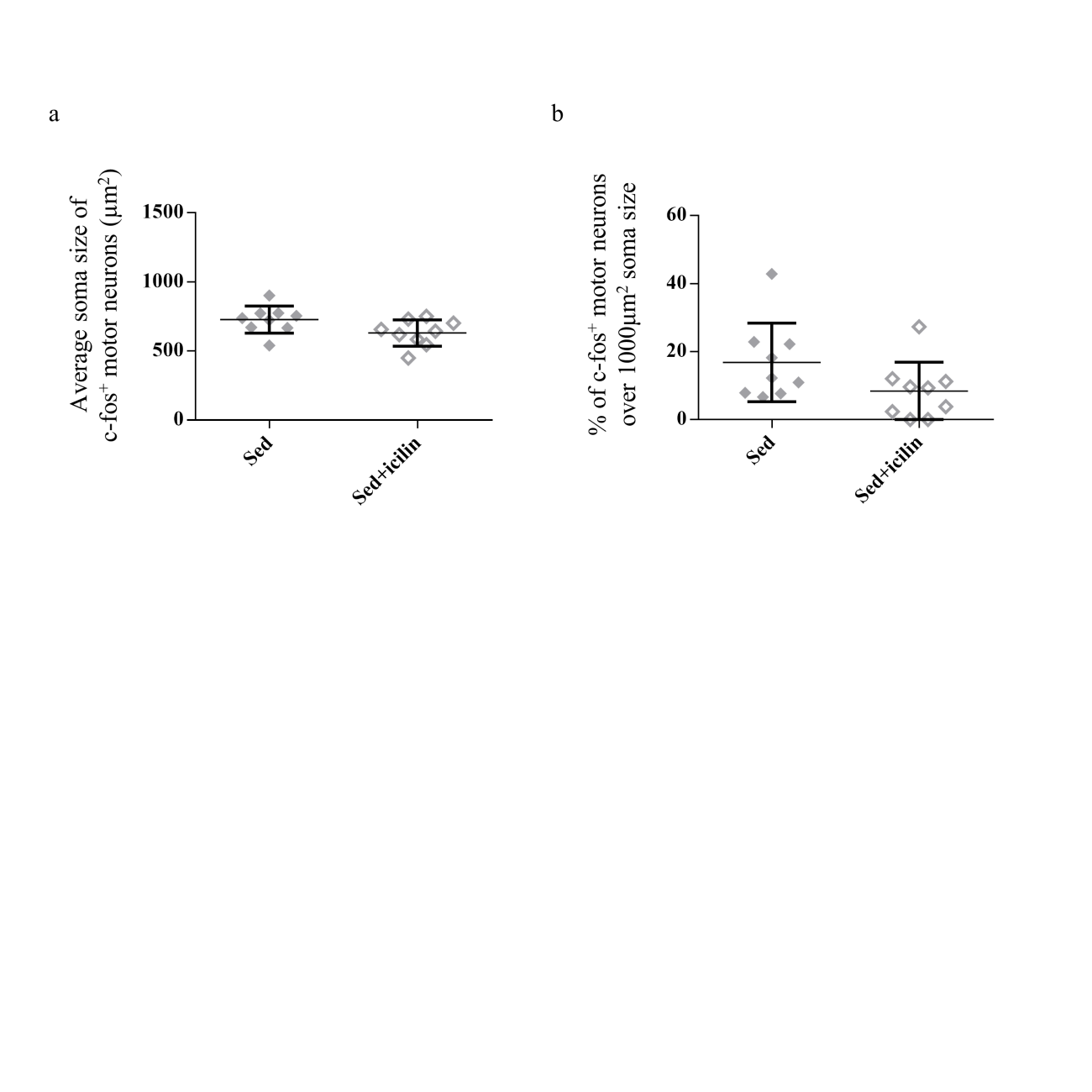

Supplement: Supplementary file 1 — Supplementary material 1 (DOCX 83 kb) [file 12576_2019_707_MOESM1_ESM.docx]
